# Sport Nutrigenomics: Personalized Nutrition for Athletic Performance

**DOI:** 10.3389/fnut.2019.00008

**Published:** 2019-02-19

**Authors:** Nanci S. Guest, Justine Horne, Shelley M. Vanderhout, Ahmed El-Sohemy

**Affiliations:** ^1^Department of Nutritional Sciences, University of Toronto, Toronto, ON, Canada; ^2^Nutrigenomix Inc., Toronto, ON, Canada; ^3^Department of Health and Rehabilitation Sciences, University of Western Ontario, London, ON, Canada

**Keywords:** nutrigenomics, nutrigenetics, personalized nutrition, athletic performance, genetic testing, sports nutrition, caffeine, ergogenic aids

## Abstract

An individual's dietary and supplement strategies can influence markedly their physical performance. Personalized nutrition in athletic populations aims to optimize health, body composition, and exercise performance by targeting dietary recommendations to an individual's genetic profile. Sport dietitians and nutritionists have long been adept at placing additional scrutiny on the one-size-fits-all general population dietary guidelines to accommodate various sporting populations. However, generic “one-size-fits-all” recommendations still remain. Genetic differences are known to impact absorption, metabolism, uptake, utilization and excretion of nutrients and food bioactives, which ultimately affects a number of metabolic pathways. Nutrigenomics and nutrigenetics are experimental approaches that use genomic information and genetic testing technologies to examine the role of individual genetic differences in modifying an athlete's response to nutrients and other food components. Although there have been few randomized, controlled trials examining the effects of genetic variation on performance in response to an ergogenic aid, there is a growing foundation of research linking gene-diet interactions on biomarkers of nutritional status, which impact exercise and sport performance. This foundation forms the basis from which the field of sport nutrigenomics continues to develop. We review the science of genetic modifiers of various dietary factors that impact an athlete's nutritional status, body composition and, ultimately athletic performance.

## Introduction

Sport and exercise performance are significantly influenced by nutrition, yet individuals respond differently to the same foods, nutrients and supplements consumed. This holds true for a variety of ages, ethnicities, and level of skill, and whether the goal is optimizing physical activity for health and fitness or for high performance sport. The importance of a personalized sports nutrition plan was highlighted in the recent “Nutrition and Athletic Performance” Joint Position Statement by the American College of Sports Medicine, the Academy of Nutrition and Dietetics and the Dietitians of Canada, which states that “Nutrition plans need to be personalized to the individual athlete… and take into account specificity and uniqueness of responses to various strategies” (1). These strategies encompass overall dietary patterns, macronutrient ratios, micronutrient requirements, eating behaviors (e.g., nutrient timing), and the judicious use of supplements and ergogenic aids.

The paradigm shift, away from the one-size-fits-all group approach and toward personalization for the individual, is moving nutrigenomics research from basic science into practice. While it has long been recognized that genetics play an influential role in determining how an athlete responds to foods and nutrients, the surge in research into gene-diet interactions over the past decade has provided a scientific basis for this hypothesis through various research initiatives and the corresponding increase in published studies. Genetic variants affect the way we absorb, metabolize, utilize and excrete nutrients, and gene-diet interactions that affect metabolic pathways relevant to health and performance are now widely recognized (2). Personal genetic testing can provide information that will guide recommendations for dietary choices that are more effective at the individual level than current dietary advice, which has been set by government agencies and other health and sport organizations. Disclosure of genetic information has also been shown to enhance motivation and behavior change and strengthen adherence to the dietary recommendations provided (2–6). Although athletes tend to exhibit higher levels of motivation in general (7), nutrition professionals still encounter significant barriers to behavior change when counseling athletes on the adoption of beneficial sports nutrition practices (8, 9). A recent systematic review found that when genetic information included actionable advice, individuals were more likely to change health behaviors, including their dietary choices and intakes (10).

**Figure 1:**
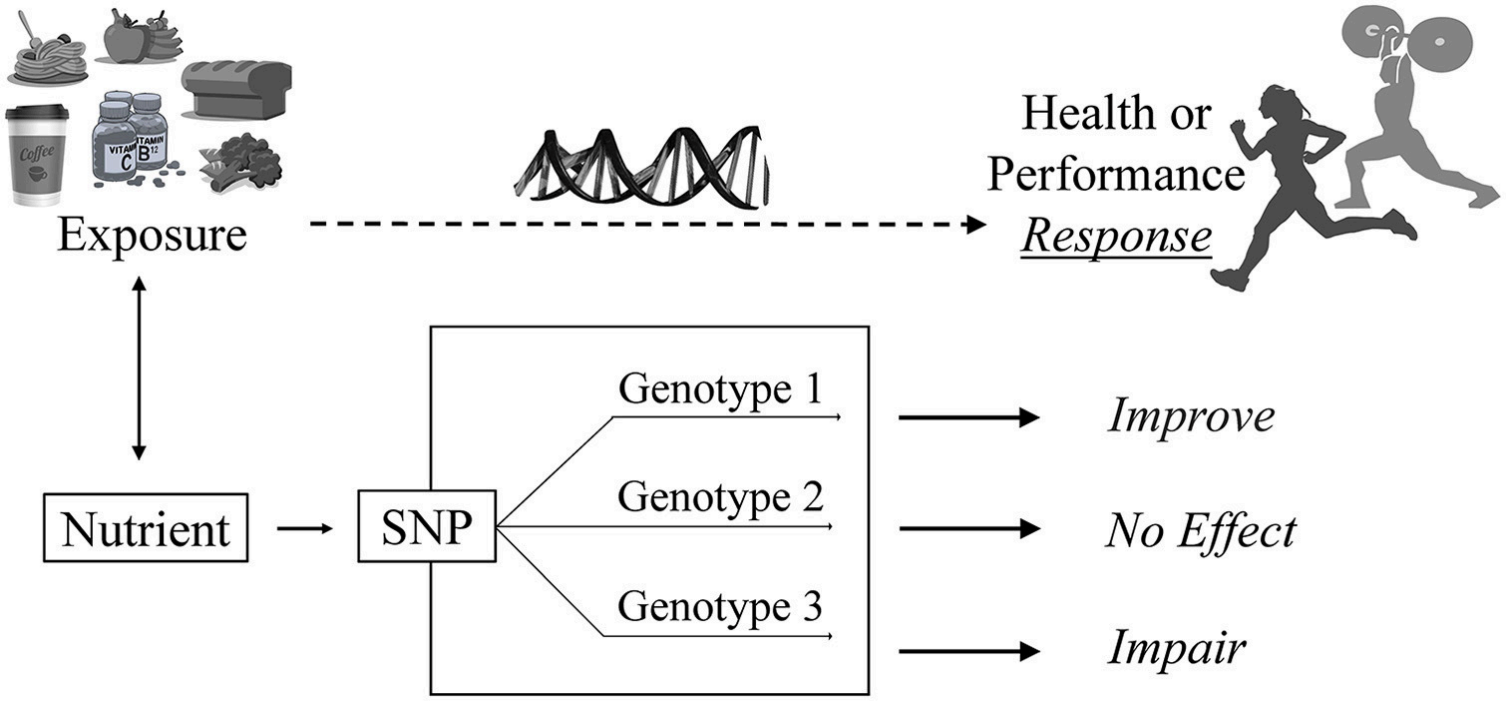
The nutrigenomics approach to sport nutrition. An athlete is exposed to a food, beverage, nutrient or bioactive. A genetic variant such as a single nucleotide polymorphism (SNP) associated with that exposure modifiers the individual's requirement for or response to that exposure. Their unique response depends on their version of the gene or “genotype.” For example, in the *CYP1A2* rs726551 SNP, individuals with the AA genotype (fast metabolizers) experience a positive or “improved” response (i.e., performance) to caffeine. Individuals with the *CYP1A2* AC or CC genotype experience no effect or impaired performance, respectively, from caffeine use (19).

The practical application of the scientific knowledge gained from research on health and performance is to enable athletes to utilize genetic test results for personalized nutrition in an actionable manner. The demand for genetic testing for personalized nutrition and associated performance outcomes by athletes and active individuals is growing, and there is an increased need for dietitian-nutritionists, fitness professionals, coaches, and other sports medicine practitioners to understand the current evidence in this developing field (11–14). The sport environment is dynamic, progressive, innovative, and extremely competitive. Providing athletes with individually tailored dietary and other performance-related information based on their DNA could yield a competitive edge. The growing body of science in nutrition and genetics is the foundational building block by which practitioners can help athletes reach their genetic potential through implementation of dietary and supplement strategies that are aligned to their genetic makeup (Figure 1). Scientific advancements along with increased interest in genetic testing have resulted in a necessary growth for professional support, where tools for proficient and knowledgeable nutrition counseling based on genetics are now more widely available. For example, the Dietitians of Canada now offer a course on “Nutrigenomics: Genetic testing for personalized nutrition” as part of their online Learning-on-Demand portal.

Personalized nutrition, based on an individual's genotype, is not a novel concept, and there are several examples of rare (e.g., phenylketonuria) and common (e.g., lactose intolerance) genetic variants that require specific dietary strategies to manage (15). Although genetic testing is well-established in the clinical setting, there is a growth in opportunities to improve health, wellness and sport performance in athletes through nutrition-focused genetic testing. In the ongoing battles against dangerous supplements (16) and unprecedented numbers of doping violations (17, 18), the sport science community is seeking novel, yet evidence-based, approaches for athletes to gain a competitive edge which are safe, effective and legal. Personalized nutrition is not limited to the identification of genetic variants. Genotype is one aspect of personal information that can be used to individualize dietary advice. An individual's genetic profile as it relates to diet should be used combination with other relevant information such as sex, age, anthropometrics, health status, family history, and socioeconomic status along with dietary preferences and the presence of food intolerances or allergies. Accompanying blood work is also useful to evaluate current nutrition status and for ongoing monitoring.

Personalized dietary and supplement advice derived from genetic testing should be based on clear and defensible interpretations of relevant research studies. Traditional genome-wide association studies (GWAS) can be used to identify associations between genotypes and outcomes of interest such as blood levels of a micronutrient. However, the utility of such markers in providing actionable information on dietary advice is limited because it is not known what dietary intakes are required to counter effects of the genetic variant(s). For example, although a genetic variant that has been associated with low serum values of a vitamin is identified, a specific recommendation for intakes to prevent the risk of deficiency or to alleviate low levels of this micronutrient may remain undetermined. Such studies require the appropriate design that demonstrates how a genetic variant modifies the response to dietary intake on the outcome trait of interest and perhaps identify responders and non-responders. Genetic markers related to a performance trait, such as aerobic capacity or power, also provide little information on what factors could be used to improve the trait of interest.

With the exception of investigations exploring genetic variation and supplemental caffeine, which have been shown to modify endurance exercise outcomes (19, 20), there are few performance studies that have examined the role of genetics and other dietary factors on athletic outcomes. A gene-diet interaction may not be associated directly with a quantifiable performance outcome, such as increased aerobic capacity, speed or strength, but rather with intermediate biomarkers or phenotypes, such as body composition or circulating vitamin D levels, which are independent determinants of athletic performance, injury-risk and post-training recovery (1, 21–24). For example, it is well-known that low iron stores impact hemoglobin production which in turn decreases the oxygen carrying capacity of the blood, leading to a lack of oxygen to working muscles and resulting in impaired muscle contraction and aerobic endurance (21). As such, genetic markers that impact iron stores in response to intake can indirectly affect performance through the oxygen carrying capacity of hemoglobin (25, 26).

### Sport Nutrigenomics Vs. Talent Identification and Exercise Prescription

In an effort to achieve specific sport goals, there is generally considerable overlap in the development of complementary training and dietary plans for athletes (27–29). However, it is essential to underscore the distinction between the strength of evidence supporting DNA-based advice for personalized nutrition vs. that for fitness programming. Despite the fervent interest and ubiquity of commercial genetic testing to assess and improve exercise or sport performance (30–32), it should be noted that there is a lack of evidence encompassing exercise prescription and talent identification, such as the ability to predict the likelihood for the next generation of Olympians (33, 34). Similarly, at this time there is insufficient evidence to recommended training protocols (strength or endurance) based on genotype or polygenic scores, that target specific fitness, weight loss or sport goals (35–38). The practical and ethical considerations of genetic testing for sports performance have also been described (39).

Some commercial genetic tests claim to use proprietary algorithmic approaches to prescribe training protocols based on evidence reported in peer-reviewed research (35). Although this may provide some initial supportive documentation for differing responses to training based on genotype, much larger sample sizes and improved methodologies are required, and should be pursued (36). The approach by which individuals are categorized as having an “endurance” or “power” advantage by genotype or being “responders” and “non-responders” to different training protocols, requires transparency and standardization across the field to avoid potential bias and to allow other researchers to replicate a study's methodology (37). Attempts to replicate studies to test training outcomes based on genotype require the use of the identical scoring systems and it appears that essential details of methods for grading of the strength of scientific evidence used in these scoring systems are not reported (35).

There is a considerable amount of ongoing research investigating individual variation in response to exercise training, however, sport and exercise genomics is still in its early stages and clinical or sport utility is lacking (36, 40–44). Mainstream testing for personalized training or exercise prescription based on genotype is not currently supported as a scientifically-sound approach, although it is likely to be a common and viably employed coaching tool within the next decade (35–37, 43, 44).

## Genes Associated With Sport Nutrition

The objective of this review is to examine the scientific evidence on specific nutrients and food bioactives whereby genetic variants appear to modify individual responses related to athlete health and athletic performance. Although many studies reviewed herein have not been studied in athletes exclusively, they have been carried out in healthy individuals. Accordingly, several studies outlined reflect optimal health, body composition and nutritional status, which for athletes, provides the foundation for athletic success. Genetic variation impacting response to various micro- and macronutrients, as well as bioactives such as caffeine, on performance-related traits will be reviewed (Table 1).

**Table 1:** Summary of Genetic Variants that modify the association between various dietary factors and performance-related outcomes.

**Gene (rs number)**	**Function**	**Dietary factor**	**Dietary sources**	**Performance-related outcome**
*CYP1A2* (rs762551)	Encodes CYP1A2 liver enzyme: metabolizes caffeine; identifies individuals as fast or slow metabolizers	Caffeine	Coffee, tea, soda, energy drinks, caffeine supplements	Cardiovascular health, endurance (21, 22, 57, 58)
ADORA2A (rs5751876)	Regulates myocardial oxygen demand; increases coronary circulation via vasodilation	Caffeine	Coffee, tea, soda, energy drinks, caffeine supplements	Vigilance when fatigued, sleep quality (49, 51–53)
BCMO1 (rs11645428)	Converts provitamin A carotenoids to Vitamin A	Vitamin A	Bluefin tuna, hard goat cheese, eggs, mackerel, carrots, sweet potato	Visuomotor skills and immunity (93, 95, 98–101)
MTHFR (rs1801133)	Produces the enzyme methylenetetrahydrofolate reductase, which is involved in the conversion of folic acid and folate into their biologically active form, L-methylfolate	Folate	Edamame, chicken liver, lentils, asparagus, black beans, kale, avocado	Megaloblastic anemia and hyperhomocysteinemia risk (112, 116–118)
HFE (rs1800562 and rs1799945)	Regulates intestinal iron uptake	Iron	Beef, chicken, fish, organ meats (heme iron); almonds, parsley, spinach (non-heme iron)	Hereditary hemochromatosis (130–132)
TMPRSS6 (rs4820268), TFR2 (rs7385804), TF (rs3811647)	Regulate the peptide hormone, hepcidin, which controls iron absorption	Iron	Beef, chicken, fish, organ meats (heme iron); almonds, parsley, spinach (non-heme iron)	Iron-deficiency anemia risk (24, 27, 120, 123–125)
FUT2 (rs602662)	Involved in vitamin B12 cell transport and absorption	Vitamin B12	Clams, oysters, herring, nutritional yeast, beef, salmon	Megaloblastic anemia and hyperhomocysteinemia (142)
GSTT1 (Ins/Del)	Plays a role in vitamin C utilization via glutathione S-transferase enzymes	Vitamin C	Red peppers, strawberries, pineapple, oranges, broccoli	Circulating ascorbic acid levels Mitigate exercise-induced ROS production (153, 155)
GC (rs2282679) and CYP2R1 (rs10741657)	GC encodes vitamin D-binding protein, involved in binding and transporting vitamin D to tissues; CYP2R1 encodes the enzyme vitamin D 25-hydroxylase involved in vitamin D activation	Vitamin D	Salmon, white fish, rainbow trout, halibut, milk	Circulating 25(OH)D levels impacting immunity, bone health, inflammation, strength training and recovery (1, 162, 164, 166, 168)
GC (rs7041 and rs4588)	GC encodes vitamin D-binding protein, involved in binding and transporting vitamin D to tissues; Vitamin D is required for calcium absorption	Calcium	Yogurt, milk, cheese, firm tofu, canned salmon (with bones), edamame	Bone/stress fracture risk Muscle contraction, nerve conduction, blood clotting (162, 164, 166, 168)
PEMT (rs12325817)	Involved in endogenous choline synthesis via the hepatic phosphatidylethanolamine *N*-methyltransferase pathway	Choline	Eggs, beef, poultry, fish, shrimp, broccoli, salmon	Muscle or liver damage, reduced neurotransmitters (174, 175, 185, 186)
MTHFD1 (rs2236225)	Encodes protein involved in trifunctional enzyme activities related to metabolic handling of choline and folate	Folate/Choline	Folate: Edamame, chicken liver, lentils, asparagus, blck beans, kale, avocado Choline: Eggs, beef, poultry, fish, shrimp, broccoli, salmon	Muscle or liver damage, reduced neurotransmitters (185, 186)
FTO (rs1558902/rs9939609)	Precise function undetermined; plays a role in metabolism and has been consistently linked to weight, BMI and body composition	Protein/SFA:PUFA	Protein: chicken, beef, tofu, salmon, cottage cheese, lentils, milk, Greek yogurt SFA: cheese, butter, red meat, baked goods PUFA: flaxseed oil, grape seed oil, sunflower oil	Optimizing body composition (190, 191)
TCF7L2 (rs7903146)	Involved in expression of body fat	Fat	Nuts/seeds, butter, oils, cheese, red meat, high-fat dairy	Optimizing body composition (192, 193)
PPARγ2 (rs1801282)	Regulates adipocyte differentiation	MUFA	Macadamia nuts, almond butter, peanut butter, olive oil, canola oil, sesame oil	Optimizing body composition (194)

### Caffeine

Caffeine, found naturally occurring in several plant species including coffee, tea, cocoa, and guarana, is widely used in sport as a performance enhancer or ergogenic aid often in the form of caffeinated tablets, gels or chews.

In the field of nutrigenomics, caffeine is the most widely researched compound with several randomized controlled trials investigating the modifying effects of genetic variation on athletic performance (19, 20, 45). Numerous studies have investigated the effect of supplemental caffeine on exercise performance, but there is considerable inter-individual variability in the magnitude of these effects (46–48), or in the lack of an effect (49, 50) when compared to placebo. These inter-individual differences appear to be partly due, to variation in genes such as *CYP1A2* and possibly *ADORA2*, which are associated with caffeine metabolism, sensitivity and response (51).

Over 95% of caffeine is metabolized by the CYP1A2 enzyme, which is encoded by the *CYP1A2* gene (52). The−163A>C (rs762551) single nucleotide polymorphism (SNP) has been shown to alter CYP1A2 enzyme activity (53–55), and has been used to identify individuals as “fast” or “slow” metabolizers of caffeine. Individuals who are considered slow metabolizers, that is with the AC or CC genotype, have an elevated risk of myocardial infarction (56), hypertension and elevated blood pressure (57, 58), and pre-diabetes (59), with increasing caffeinated coffee consumption, whereas those with the AA genotype (fast metabolizers) do not appear to carry these risks.

The largest caffeine and exercise study to date (19), examined the effects of caffeine and *CYP1A2* genotype, on 10-km cycling time trial performance in competitive male athletes after ingestion of caffeine at 0 mg, 2 mg (low dose) or 4 mg (moderate dose) per kg body mass. There was a 3% improvement in cycling time in the moderate dose in all subjects, which is consistent with previous cycling time trial studies using similar doses (46, 60). However, there was a significant caffeine-gene interaction where improvements in performance were seen at both caffeine doses, but only in those with the AA genotype who are “fast metabolizers” of caffeine. In that group, a 6.8% improvement in cycling time was observed at 4 mg/kg, which is >2–4% mean improvement seen in several other cycling time trial studies, using similar doses (46, 60–65). Among those with the CC genotype, 4 mg/kg caffeine impaired performance by 13.7%, and in those with the AC genotype there was no effect of either caffeine dose (19). The findings are consistent with a previous study (20), which observed a caffeine-gene interaction and improved time trial cycling performance with caffeine only in those with the AA genotype.

Some previous endurance-type studies either did not observe any impact of the *CYP1A2* gene on caffeine-exercise studies (66, 67), or reported benefits only in slow metabolizers (45). There are several reasons that may explain discrepancies in study outcomes including smaller sample sizes (<20 subjects) that cause very low numbers and/or no subjects with the CC genotype (45, 67, 68), and shorter distance or different type (power vs. endurance) of performance test (45), compared to those that reported improved endurance after caffeine ingestion in those with the AA genotype of *CYP1A2* (19, 20). The effects of genotype on performance appear to be most prominent during exercise of longer duration or an accumulation of fatigue (aerobic or muscular endurance) (69, 70). Fast metabolizers may quickly metabolize caffeine and achieve the benefits of caffeine metabolites as exercise progresses, or override the short duration of negative impacts (the initial stages of exercise), whereas the adverse effects of restricted blood flow and/or other impacts of adenosine blockage in slow metabolizers are likely to remain for a longer duration (71, 72). Indeed, in a study of basketball performance in elite players, caffeine improved repeated jumps (muscular endurance; an accumulation of fatigue), but only in those with the AA genotype, however, there was no genotype effect in the other two performance components of the basketball simulation (73). Similarly, a cross-over design of 30 resistance-trained men found that caffeine ingestion resulted in a higher number of repetitions in repeated sets of three different exercises, and for total repetitions in all resistance exercises combined, which resulted in a greater volume of work compared to placebo conditions, but only in those with the *CYP1A2* AA genotype (74). Taken together, the weight of the evidence supports the role of *CYP1A2* in modifying the effects of caffeine ingestion on aerobic or muscular endurance-type exercise.

The *ADORA2A* gene is another potential genetic modifier of the effects of caffeine on performance. The adenosine A_2A_ receptor, encoded by the *ADORA2A* gene, has been shown to regulate myocardial oxygen demand and increase coronary circulation by vasodilation (71, 72). The A_2A_ receptor is also expressed in the brain, where it regulates glutamate and dopamine release, with associated effects on insomnia and pain (75, 76). The antagonism of adenosine receptors by caffeine could differ by *ADORA2A* genotype, resulting in altered dopamine signaling (51). Dopamine has been associated with motivation and effort in exercising individuals, and this may be a mechanism by which differences in response to caffeine are manifested (77–79).

One small pilot study has examined the effect of *ADORA2A* genotype (rs5751876) on the ergogenic effects of caffeine under exercise conditions (80). Twelve female subjects underwent a double-blinded, crossover trial comprising two 10-min cycling time trials following caffeine ingestion or placebo. Caffeine benefitted all six subjects with the TT genotype but only one of the six C allele carriers. Further studies are needed to confirm these preliminary findings and include a larger sample to distinguish any effects between the different C allele carriers (i.e., CT vs. CC genotypes).

Sleep is recognized as an essential component of physiological and psychological recovery from, and preparation for, high-intensity training in athletes (81, 82). The *ADORA2A* rs5751876 genotype has also been implicated, by both objective and subjective measures, in various parameters of sleep quality after caffeine ingestion in several studies (83–86). Adenosine promotes sleep by binding to its receptors in the brain, mainly A_1_ and A_2A_ receptors, and caffeine reverses these effects by blocking the adenosine receptor, which promotes wakefulness (83). This action, as well as the potency of caffeine to restore performance (cognitive or physical) in ecological situations, such as highway-driving during the night (87), support the notion that the adenosine neuromodulator/receptor system plays a major role in sleep–wake regulation. This action of caffeine may also serve athletes well under conditions of jetlag, and irregular or early training or competition schedules. Psychomotor speed relies on the ability to respond, rapidly and reliably, to randomly occurring stimuli which is a critical component of most sports (88). Genetic variation in *ADORA2A* has been shown to be a relevant determinant of psychomotor vigilance in the rested and sleep-deprived state and modulates individual responses to caffeine after sleep deprivation (85). In support of this notion, individuals who had the TT genotype for *ADORA2A* rs5751876 consistently had faster response times (in seconds) than C allele carriers after ingesting 400 mg caffeine during a sustained vigilant attention task after sleep loss (85).

Consistent with the “adenosine hypothesis” of sleep where the accumulation of adenosine in the brain promotes sleep, caffeine prolongs the time to fall asleep, decreases the deep stages of non-rapid-eye movement (nonREM) sleep, reduces sleep efficiency, and alters the waking and sleep electroencephalogram (EEG) frequencies, which reliably reflect the need for sleep (89–91). Although additional research in this area is warranted, genetic variation appears to contribute to subjective and objective responses to caffeine on sleep. Carriers of the *ADORA2A* (rs5751876) C allele have greater sensitivity toward caffeine-induced sleep disturbance compared to those with the TT genotype (84). Taken together, it appears that individuals with the TT genotype for the rs5751876 SNP in the *ADORA2A* gene may have better performance outcomes, faster response times and less sleep disturbance following caffeine ingestion.

### Vitamin A

No studies have examined the role of genetic modifiers of vitamin A status directly on athletic performance, however, there are several important functions of this micronutrient that are associated with optimal health, immunity and performance in athletes.

Vitamin A is a fat-soluble vitamin, which plays a key role in both vision (92) and immunity (93) in its biologically active forms (retinal and retinoic acid). Vitamin A has diverse immune modulatory roles; hence, vitamin A deficiency has been associated with both immune dysfunctions in the gut, and several systemic immune disorders (93). Vitamin A is also a powerful antioxidant, protecting eyes from ocular diseases and helping to maintain vision (92).

High-performance athletes appear to have superior visual abilities based on their capacity to access distinct visual skills, such as contrast sensitivity, dynamic acuity, stereoacuity, and ocular judgment, needed to accomplish interceptive actions (e.g., hand-eye coordination) and resolve fine spatial detail, which is required by many sports (94, 95). In addition, slow visuomotor reaction time (VMRT) has been associated with musculoskeletal injury risk in sporting situations where there are greater challenges to visual stimulus detection and motor response execution (96). These visuomotor skills are key contributors to enhanced sport performance, and accordingly, require exceptional eye health.

Deficiencies of certain micronutrients such as vitamin A decrease immune defense against invading pathogens and can cause the athlete to be more susceptible to infection. Low energy availability (dieting), poor food choices, jetlag, physical and psychological stress, and exposure to pollution and foreign pathogens in air, food and water while traveling can result in a deterioration in immune function and increased susceptibility to illness (97). Athletes following high volume, high intensity training and competition schedules are also known to have more frequent upper respiratory tract infections (URTI) compared to both sedentary and moderately exercising populations (97).

Upon absorption, provitamin A carotenoids are readily converted to vitamin A by the BCMO1 enzyme expressed in enterocytes of the intestinal mucosa (98). β-Carotene is the most abundant provitamin A carotenoid in the diet and the conversion of beta-carotene to retinal or retinoic acid is necessary for vitamin A to exert its biological functions. The rs11645428 variant in the *BCMO1* gene affects circulating plasma carotenoid levels by impacting the conversion of dietary provitamin A carotenoids to active forms of vitamin A in the small intestine (99). Individuals with the GG genotype are inefficient at this conversion, and may be at higher risk for vitamin A deficiency (100). These individuals are considered low responders to dietary β-carotene so consuming enough dietary pre-formed vitamin A (or supplements for vegans), can help to ensure that circulating levels of active vitamin A are adequate to support vision, immunity and normal growth and development.

### Anemia-Related Micronutrients: Iron, Folate, and Vitamin B_12_

There is an abundance of research demonstrating the adverse effects of low iron storage and anemia on athletic performance (23, 101–103). The estimated prevalence of anemias and low levels of iron, folate, and vitamin B_12_ appear to be higher in elite-level athletes than in the general population, and these deficiencies can have significant negative impacts on performance (22, 23, 104–107). The most common symptoms of this disorder are fatigue, weakness and, in extreme cases, shortness of breath or palpitations (103).

The importance of iron to athletes is established through its biological role in supporting the function of proteins and enzymes essential for maintaining physical and cognitive performance (108). Iron is incorporated into hemoglobin and myoglobin, proteins responsible for the transport and storage of oxygen. Iron-deficiency anemia is the most common type of anemia among athletes, who have higher iron requirements due to increased erythropoietic drive through higher intensities and volumes of training. The female athlete is at particular risk of iron deficiency due to menstruation and generally, a lower total energy or food intake compared to males (107, 109). Along with dietary intake, footstrike hemolysis, gastrointestinal bleeding, exercise-induced inflammation, non-steroidal autoinflammatory drug (NSAID) use and environmental factors such as hypoxia (altitude), may influence iron metabolism in athletes of both sexes (23). Macrocytic anemias, which occur when erythrocytes are larger than normal, are generally classified into megaloblastic or nonmegaloblastic anemia. Megaloblastic anemia is caused by deficiency or impaired utilization of vitamin B_12_ and/or folate, whereas non-megaloblastic macrocytic anemia is caused by various diseases, and will not be discussed here (110). Other factors that are associated with anemia risk include genetic variation, which can alter micronutrient metabolism, transport or absorption, and can be used to identify individuals at risk of inadequate levels of vitamin B_12_, folate and iron stores.

Performance improvements are usually seen with the treatment of anemia (23, 103, 104), which is related to improvements in symptoms such as general feelings of fatigue and weakness, difficulty exercising, and in more severe cases, dyspnea and palpitations (103). Hyperhomocysteinemia, which can result from low folate and/or vitamin B_12_ intake, may also increase the risk of skeletal muscle malfunction, including muscle weakness and muscle regeneration, and will be discussed further below (111).

#### Folate

Methylene tetrahydrofolate reductase (MTHFR) is the rate-limiting enzyme in the methyl cycle, and is encoded by the *MTHFR* gene (112). The C677T (rs1801133) polymorphism in the *MTHFR* gene has been associated with low serum and red blood cell folate as well as elevated plasma homocysteine levels, which is an independent risk factor for cardiovascular disease (CVD) (113, 114). Several studies in athletic and non-athletic populations have shown that individuals with the CT or TT genotype are at an increased risk of low circulating folate levels when their diet is low in folate (115–118).

Although there are no studies examining performance outcomes related to *MTHFR* genotypes or dietary folate intake, hyperhomocysteinemia has been shown to be associated with diminished muscle function (111). Several studies conducted in older adults have found a significant association between elevated plasma homocysteine concentrations and declined physical function (119–122), which may be mediated by a reduction in strength (120). Compared to those with the rs1801133 CC genotype, individuals with TT genotype and possibly the CT genotype may be at a greater risk for hyperhomocysteinemia, although this may not be causative for lower physical performance (111, 119, 120). However, soccer players and sedentary individuals with the CC genotype have been shown to have more favorable body composition and performance measures such as aerobic and anaerobic threshold rates, compared to carriers of the T allele (118).

#### Iron Overload

Genetic variation associated with serum iron levels involves several genes such as *HFE, TMPRSS6, TFR2*, and *TF* (25, 117, 123–128). The *HFE* gene is involved in the regulation of intestinal iron uptake (129), and variations in this gene, which are not very common, have been shown to increase the risk for hemochromatosis or iron overload (124, 130). Excess iron may be toxic to tissues and cells because highly reactive “free” iron reacts with reactive oxygen species (ROS) such as superoxide and hydrogen peroxides, or lipid peroxides to produce free radicals (131). In turn, these free radicals can cause cell and tissue damage (including muscle) and, ultimately, lead to cell death (132). Elevated biomarkers of iron such as ferritin and transferrin are more common in those who are genetically predisposed to iron overload based on the *HFE* gene variant (22, 124). Interestingly, athletes with the rare *HFE* (rs1800562) AA genotype, which is associated with an increased risk for hemochromatosis, may be at a genetic advantage to excel in sport if iron levels are at the high end of the normal range, but not excessive to cause tissue damage. Notably, several studies have found that certain variants of the *HFE* gene that increase risk of iron overload are more common in elite-level athletes compared to the general population, suggesting this may be beneficial for performance (133–135).

Two SNPs in the *HFE* gene (rs1800562 and rs1799945) can be used to predict risk of hereditary hemochromatosis. Based on the combination of variants from these two SNPs, individuals can be categorized as having a high, medium, or low risk for iron overload (124, 128). While genetic risk for iron overload may have a favorable impact on performance, it is necessary for athletes with a medium or high risk to avoid iron supplementation as this could lead to adverse health outcomes (124) and diminished performance.

#### Low Iron Status

Three main SNPs: *TMPRSS6* (rs4820268), *TFR2* (rs7385804), *TF* (rs3811647) can be used to assess genetic risk for low iron status, primarily due to their involvement in regulating the expression of hepcidin, which is a peptide hormone that controls iron absorption (25, 123, 127). Iron-deficiency anemia impairs performance by reducing oxygen-carrying capacity, but a number of reports indicate that iron deficiency without anemia may affect physiological performance and work capacity as well (21), particularly in women who experience iron deficiency more frequently (22, 23).

There is a fine balance in achieving and maintaining adequate, but not excessive, iron levels for optimal performance. Individuals with the GG genotype in the *TMPRSS6* gene have an increased risk of low transferrin saturation and hemoglobin, compared to those who are carriers of the A allele (25, 26, 123, 136). In the *TF* gene, individuals have a greater risk for low ferritin and elevated transferrin when they possess the AA genotype (25, 123, 136). Variation in the *TFR2* gene can impact hematocrit, mean corpuscular volume, and red blood cell count where individuals with the CC genotype have an increased risk of low serum levels (25). Utilizing algorithms to assess various genotype combinations, these genes can help to determine an individual's overall risk for low iron status, which can lead to iron-deficiency anemia, and can be used to target dietary iron intake. Although iron supplementation is common and frequently prescribed in athletes, many individuals are at risk of taking iron supplements in excess (22, 137, 138). Although iron supplements are commonly “prescribed” by healthcare professionals and nutritionists (139, 140), excess iron stored in skeletal muscle may not only be dangerous to the health of the athlete (124, 141), but also can lead to oxidative stress and the formation of free radicals, and reduced athletic performance (135, 142, 143).

#### Vitamin B_12_

Vitamin B_12_ is also associated with RBC formation and aerobic capacity. Megaloblastic anemia results from vitamin B_12_ deficiency and is associated with elevated homocysteine, and results in general feelings of fatigue and weakness. Megaloblastic anemia limits the blood's oxygen carrying capacity, thus reducing its availability to cells (144). Variation in the *FUT2* gene (rs602662) has a significant impact on serum B_12_ levels where individuals with GG or GA genotypes possess the greatest risk for low serum vitamin B_12_ levels, but only when the diet is low in bioavailable sources of vitamin B_12_ (145). This is consistent with previous genome-wide association studies, which found that individuals with the AA genotype had significantly higher concentrations of serum vitamin B_12_ compared to carriers of the G allele (145).

### Vitamin C

Vitamin C is a water-soluble antioxidant that aids in the reduction of exercise-induced free-radical production (146). The production of potentially harmful ROS (147–149) in athletes is greater than in non-athletes due to the massive increases (up to 200-fold at the level of skeletal muscle) in oxygen consumption during strenuous exercise (146, 150). Vitamin C supplementation was once thought to mitigate this risk; however, studies have shown that excess vitamin C supplementation during endurance training can blunt beneficial training-induced physiological adaptations, such as muscle oxidative capacity and mitochondrial biogenesis and may actually diminish performance (148, 149, 151, 152). Dietary consumption of vitamin C, up to 250 mg daily from fruits and vegetables, is likely sufficient to reduce oxidative stress without having a negative effect on performance (151, 153). Additionally, collagen is a key constituent of connective tissue such as tendons and ligaments, and vitamin C is necessary for collagen production. This suggests that vitamin C may play a role in muscle growth and repair (154, 155). Indeed, a recent landmark study examining collagen synthesis in athletes, reported that adding a gelatin and vitamin C supplement to an intermittent exercise protocol improves collagen synthesis and could play a beneficial role in injury prevention and accelerate musculoskeletal, ligament, and/or tendon tissue repair (155).

The relationship between dietary vitamin C and circulating levels of ascorbic acid depend on an individual's *GSTT1* genotype (156). Individuals who do not meet the Recommended Dietary Allowance (RDA) for vitamin C are significantly more likely to be vitamin C deficient (as assessed by serum ascorbic acid levels) than those who meet the RDA, but this effect is much greater in individuals with the *GSTT1* Del/Del genotype than those with the Ins allele (156).

Genetic testing can help to identify athletes who may be at the greatest risk of low circulating vitamin C (ascorbic acid) levels in response to intake. These low circulating ascorbic acid levels may, in turn, diminish performance through an increased risk of high ROS and diminished muscle or connective tissue repair. Although studies have identified associations between circulating ascorbic acid concentrations and vitamin C transporters, SVCT_1_ and SVCT_2_, which are encoded by *SLC23A1* and *SLC23A2* (157), there is no evidence that response to vitamin C intake differs by genotype (158). As such, the use of variants in *SLC23A1* and *SLC23A2* to make personalized dietary recommendations is not supported by the studies to date.

### Vitamin D

There are no studies that link genetic modifiers of vitamin D status on athletic performance outcomes; however, there are several functions of this vitamin that are associated with bone health, immunity, recovery from training and various performance variables. Genetic determinants of circulating 25-hydroxyvitamin D (25(OH)D) can influence each of these factors thereby influencing performance.

Vitamin D is essential to calcium metabolism, increasing calcium absorption for optimal bone health (1), which is relevant to all athletes, but particularly those participating in sports with a high risk of stress fracture (159–161). Research comparing individuals with sufficient levels to insufficient or deficient levels of 25(OH)D has shown that it helps to prevent injury (159–161), promote larger type II muscle fiber size (24), reduce inflammation (162), reduce risk of acute respiratory illness (159, 160) enhance functional rehabilitation (162), thereby optimizing recovery and acute adaptive responses to intense training through reduced inflammation and increased blood flow (163, 164).

Two genes that have been shown to impact vitamin D status are the *GC* gene and the *CYP2R1* gene (165, 166). Variations in the *GC* and *CYP2R1* genes are associated with a greater risk for low serum 25(OH)D. In one study (165), where 50% of participants took vitamin D supplements, only 22% of the participants had sufficient serum 25(OH)D levels. In the remaining 78% who had insufficient levels, also only about half (47%) took vitamin D supplements. Within this population, vitamin D supplementation only explained 18% of the variation, compared to 30% from genetics, suggesting that genetics may play a greater role than supplementation in determining risk for low 25(OH)D levels (165). Out of the four genotypes analyzed, only *CYP2R1* (rs10741657) and *GC* (rs2282679) were significantly associated with vitamin D status. Specifically, participants with the GG or GA genotype of *CYP2R1* (rs10741657) were nearly four times more likely to have insufficient vitamin D levels. Those with the GG genotype of the *GC* gene (rs2282679) were significantly more likely to have low vitamin D levels compared to those with the TT genotype (165). These results were consistent with findings from previous studies, including the Study of Underlying Genetic Determinants of Vitamin D and Highly Related Traits (SUNLIGHT), which found significance on a genome-wide basis in 15 cohorts with over 30,000 participants between three genetic variants including *CYP2R1* (rs10741657) and *GC* (rs2282679) on vitamin D status. Not surprisingly, the number of risk variants that the participants possessed was directly related to their risk for vitamin D insufficiency (166). These findings demonstrate that genetic variation may be more impactful than supplementation intakes and behaviors on determining risk for vitamin D insufficiency.

### Calcium

Although studies linking calcium intake, genetics and bone fracture has not been conducted in athletes specifically, genetic variation as it relates to risk of calcium deficiency and fracture risk have been studied in a large cohort of individuals, described below (167). Calcium is necessary for growth, maintenance and repair of bone tissue and impacts maintenance of blood calcium levels, regulation of muscle contraction, nerve conduction, and normal blood clotting (168). In order to absorb calcium, adequate vitamin D intake is also necessary. Inadequate dietary calcium and vitamin D increases the risk of low bone mineral density (BMD) and stress fractures. Low energy intakes, and menstrual dysfunction in female athletes, along with low vitamin D and calcium intakes further increase the risk of stress fractures in both males and females (169–171), and stress fractures are common and serious injuries in athletes (172).

Some individuals do not utilize dietary calcium as efficiently as others and this may depend on variations in the *GC* gene. In one study (167), subjects (*n* = 6,181) were genotyped for two SNPs in the *GC* gene, rs7041 and rs4588, and calcium intake was assessed in relation to the participants' risk for bone fracture (167). In the entire sample of participants, only a small increased risk of bone fracture was observed for individuals homozygous for the G allele of *GC* (rs7041) and the C allele of *GC* (rs4588). However, in participants with low dietary calcium intake (<1.09 g/day) and who were homozygous for the G allele of rs7041 and the C allele of rs4588, there was a 42% increased risk of fracture compared to other genotypes. No differences between genotypes were found in participants with high dietary calcium intakes (167). These findings suggest that calcium intake recommendations could be based on *GC* genotype in athletes to help prevent stress fracture.

### Choline

Choline was officially recognized as an essential nutrient by the Institute of Medicine (IOM) in 1998 (173). Choline plays a central role in many physiological pathways including neurotransmitter synthesis (acetylcholine), cell-membrane signaling (phospholipids), bile and lipid transport (lipoproteins), and methyl-group metabolism (homocysteine reduction) (174). Human requirements for choline are dependent on gender, age and physical activity level as well as genetics. Choline is produced in the body in small amounts, however, *de novo* synthesis of choline alone is not sufficient to meet human requirements for optimal health (174, 175). The liver and muscles are the major organs for methyl group metabolism, and choline deficiency has been shown to cause both liver and muscle damage (176, 177). Signs of choline deficiency are identified through elevated serum creatine phosphokinase (CPK), a marker of muscle damage (178, 179), and abnormal deposition of fat in the liver, which may result in non-alcoholic fatty liver disease (NAFLD) (176, 180). Reductions in plasma choline associated with strenuous exercise such as triathlons and marathon running have been reported (181, 182). Acetylcholine, a neurotransmitter involved in learning, memory, and attention, depends on adequate choline and a reduction in the release of this neurotransmitter may contribute to the development of fatigue and exercise performance impairment (181–183). Choline supplementation may also improve lipid metabolism, as it has been associated with more favorable body composition (184) and the ability to aid rapid body mass reduction in weight class sports (185).

Common genetic variants in choline (*PEMT gene*) and a folate pathway enzyme *(MTHFD1)* have been shown to impact the metabolic handling of choline and the risk of choline deficiency across differing nutrient intakes (178, 186, 187). The relationship between genetic variants in folate metabolism and choline requirement may arise from the overlapping roles of folate and choline in methionine and phosphatidylcholine (PC) biosynthesis. PC is critical for the structural integrity of cell membranes and cell survival, and methionine is an essential amino acid that plays a critical role in human metabolism and health (187, 188). The *MTHFD1* rs2236225 SNP, which is associated with folate metabolism, has been shown to increase the demands for choline as a methyl-group donor, thereby increasing dietary requirements for this nutrient (188). Individuals that are A allele carriers of the *MTHFD1* gene have been shown to develop signs of choline deficiency and organ (liver and muscle) dysfunction compared to those with the GG genotype (186, 188, 189).

While humans can make choline endogenously *via* the hepatic phosphatidylethanolamine *N*-methyltransferase (*PEMT*) pathway, a SNP in the *PEMT* gene (rs12325817) has been shown to influence the risk of choline deficiency and the partitioning of more dietary choline toward PC biosynthesis at the expense of betaine synthesis (used a methyl donor) (186). Individuals who are C allele carriers of the *PEMT* gene have been shown to develop signs of choline deficiency and organ (liver and muscle) dysfunction compared to those with the GG genotype (178).

Athletes by nature experience muscle damage through high volume and high intensity training (195). A deficient or suboptimal status of choline may place additional stressors on an athlete's ability to recover, repair and adapt to their given training stimulus.

### Macronutrients and Body Composition

Several aspects of physique such as body size, shape and composition contribute to the success of an athlete, in most sports. In the athletic population, body composition is often the focus for change, as it can be easily manipulated through diet as both total energy intake and macronutrient composition (192, 196). Variations in macronutrient intake can significantly impact both body fat percentage and lean mass (29, 190, 193, 197–199), as well as performance, where macronutrient manipulation has long been used to partition calories to be used for specific goals across different sports (196).

Although research examining dietary factors and genetics has revealed that manipulation of dietary fat and protein intakes may have greater modifying effects on body composition than carbohydrates, all macronutrients serve a critical purpose. Carbohydrates provide a key fuel for the brain, CNS and working muscles, and the amount and timing of intake impacts sport performance over a large range of intensities (200, 201). Adequate dietary protein is essential for strength and lean body mass accretion, while also playing a relevant role in preserving lean body mass during caloric restriction and immune function (29, 202, 203). Dietary fat provides energy for aerobic activities and is required for the absorption of fat-soluble vitamins (204). Recent research shows that percent energy intake from protein and fat can be targeted to the individual based on genetic variation for optimizing body weight and composition (190, 193, 197–199). Percent energy from carbohydrates should be guided by fuel needs for training and competition while also considering targeted protein and fat intakes based on genetic variation.

#### Protein

The *FTO* gene is also known as the ‘fat mass and obesity-associated gene’ since it has been shown to impact weight management and body composition (194, 199, 205, 206). Dietary interventions may mitigate genetic predispositions associated with a higher body mass index (BMI) and body fat percentage, as determined by genetic variation in the *FTO* gene. Specifically, the Preventing Overweight Using Novel Dietary Strategies (POUNDS Lost) multicenter trial found that carrying an A allele of the *FTO* gene (rs1558902–a surrogate marker for rs9939609) and consuming a high protein diet was associated with a significantly lower fat mass at the 2-year follow up period compared to carrying two T alleles. Importantly, participants with the AA genotype (lesser effects in those with AT genotype) who were following the high protein diet protocol had significantly greater losses of total fat mass, total adipose tissue, visceral adipose tissue, lower total percent fat mass and percent trunk fat, compared to those following a lower protein diet protocol (199). Other studies have shown similar results where dietary protein intake was shown to be protective against the effect of the *FTO* risk variants on BMI and waist circumference (194). A randomized controlled trial (RCT) in 195 individuals showed that a hypocaloric diet resulted in greater weight loss in rs9939609 A allele carriers than noncarriers in both higher and lower protein diets, although metabolic improvements improved in all genotypes in the higher protein diets (205). Athletes who possess the AA genotype of the *FTO* gene at rs1558902 would benefit the most in terms of consuming a moderate-to-high protein diet (at least 25% of energy from protein) to optimize body composition. Greater lean mass in athletes has been associated with improved performance in strength and power sports, as well as some endurance events, and a decreased risk for injuries (191, 207). For those athletes who do not possess the response variant (i.e., greater fat loss with higher protein intakes), following a diet with moderate protein intake (~15–20% energy), to achieve and maintain an ideal body composition is important to note, as excess protein calories may be counterproductive toward this goal. In this instance, dietary goals for optimal performance may be better met by substituting protein energy for other macronutrients such as carbohydrates for fuel, fiber, prebiotics and other micronutrients, or by increasing intakes of essential fats.

#### Dietary Fat

Dietary fat, an essential component of the human diet, provides energy for aerobic endurance exercise and is necessary for the absorption of the fat-soluble vitamins A, D, E, and K. Independent of total energy intake, the percentage of energy derived from fat in an athlete's diet can impact body composition, based on genetic variation (204). Individuals possessing the TT genotype of *TCF7L2, transcription factor 7 like 2*, at rs7903146 appear to benefit from consuming a lower percent of total energy from fat (20–25% of energy) to optimize body composition (198). Specifically, participants with the TT genotype lost more fat mass when they were consuming a low-fat diet, compared to a high-fat diet (40–45% of energy) (198). Moreover, individuals with the CC genotype in rs7903146 who consumed lower-fat diets actually lost significantly more lean mass, suggesting that these individuals should avoid low-fat nutrition interventions (197) in order to optimize body composition for athletic performance (191, 207). Body composition can, therefore, be optimized by targeting fat intake based on genetic variation in the *TCF7L2* gene.

#### Monounsaturated Fat

Recommendations for fat intake can be further targeted to the different types of fats comprising total dietary fat. Athletes with the GG or GC genotype of the *PPAR*γ*2* gene at rs1801282 would benefit from a weight loss intervention that specifically targets body fat, while preserving lean body mass. Such individuals have been shown to demonstrate an enhanced weight loss response when consuming > 56% of total fat from monounsaturated fatty acids (MUFAs) compared to those with the GG or GC genotype who consume < 56% of total fat from MUFAs. These results have not been found in those with the CC genotype of *PPAR*γ*2* at rs1801282 (208).

MUFAs can be targeted in athletes who are aiming to decrease their body fat. It is well-known that a lower body fat percentage is associated with enhanced performance in most sports (191, 207), however, sport clinicians must be cautious about nutrition recommendations aimed at reducing body fat. Striving for very low levels of body fat is highly correlated with the Relative Energy Deficiency in Sport (RED-S) syndrome in both females and males, which refers to ‘impaired physiological functioning caused by relative energy deficiency and includes impairments of metabolic rate, menstrual function, bone health, immunity, protein synthesis and cardiovascular health (209).

#### Saturated Fat and Polyunsaturated Fat

A nested case-control study found that the ratio of dietary saturated fatty acids (SFA) to polyunsaturated fatty acids (PUFA) influenced the risk of obesity associated with the TA and AA variants of the *FTO* gene at rs9939609 (210). Specifically, participants possessing the A allele had a significantly higher BMI and waist circumference (WC) compared to TT homozygotes, but only when intakes of SFA were high and PUFAs were low. When participants with the A allele consumed < ~15% of energy from SFA and had a higher dietary PUFA:SFA ratio, there were no significant differences in WC and BMI between this group and participants with the TT genotype of rs9939609 (210). These findings have implications for nutrition counseling impacting body composition (abdominal fat specifically) and BMI. Athletes with the TA or AA genotype may have a greater risk for accumulating excessive abdominal fat. An athlete can mitigate this risk by aiming to consume <10% of energy from SFA (to also account for heart health) and > 4% of energy from PUFAs, resulting in a PUFA:SFA ratio of at least 0.4 (210).

## Summary

This paper provided an overview of the current science linking genetic variation to nutritional or supplemental needs with a focus on sport performance. One of the ultimate goals in the field of personalized sport nutrition is the design of tailored nutritional recommendations to improve direct and indirect factors that influence athletic performance. More specifically, personalized nutrition pursuits aim to develop more comprehensive and dynamic nutritional and supplement recommendations based on shifting, interacting parameters in an athlete's internal and external (sport) environment throughout their athletic career and beyond.

Currently, there are few gene-diet interaction studies that have directly measured performance outcomes and been conducted in competitive athletes, so this should be a focus of future research. However, it has been established that serum levels and/or dietary intakes of several nutrients and food bioactives can impact overall health, body composition and in turn result in modest to sizable modifying effects in athletic performance. The strongest evidence to date appears to be for caffeine on endurance performance with several trials demonstrating the modifying effects of genetic variants with sports performance outcomes. Genetic testing for personalized nutrition may, therefore, be an additional tool that can be implemented into the practice of sport clinicians, nutritionists and coaches to guide nutritional counseling and meal planning with the aim of optimizing athletic performance.

## Author Contributions

NG and AE-S conceived of the original idea for the review and NG wrote the first draft. All authors contributed to the writing and editing of the manuscript. AE-S secured funding.

### Conflict of Interest Statement

AE-S is the Founder and holds shares in Nutrigenomix Inc. NG is on the Science Advisory Board of Nutrigenomix Inc. SV was a paid employee and remains a paid consultant with Nutrigenomix. The remaining author declares that the research was conducted in the absence of any commercial or financial relationships that could be construed as a potential conflict of interest.
